# The Story of the Insane from Year to Year

**Published:** 1890-09-06

**Authors:** 


### The Story of the Insane from Year to Year.

[Annual Reports, Pamphlets, &c., should be addressed to The Editor, The Lodge, Porchester Square, London, W.]

### THE BROOKWOOD ASYLUM.

This is now the only asylum for the County of Surrey, those at Cane Hill and Wandsworth having been taken over by the County of London under the Local Government Act. On the last day of the year there were 1,050 patients in residence. During the year 284 patients were admitted; 94 recovered, and 80 died. Put into percentages these numbers yield:— Recoveries, calculated on admissions, 46·76. Deaths, calculated on the average number resident, 7·61. There were two cases of typhoid, and in both instances the disease was imported. Apart from these there was an immunity from epidemic disease. The printer attendant received a pension of £26 a-year after seventeen years' service. The Commissioners say they "found the asylum in its usual excellent order," and the committee "are satisfied that everything is done to promote the welfare and comfort of the patients." The maintenance rate was 8s. 9d. a-week per head during the first part of the year, and 9s. 6d. during the second half.

### THE DERBY COUNTY ASYLUM.

Dr. Lindsay's report is as usual an interesting one. He deals with most subjects pertaining to asylum management, and we find paragraphs on diet, attendants' wages, amusements, occupations, water supply, and pensions of asylum officials. In the latter question Dr. Lindsay has long taken a very active interest, and it is to be hoped that some definite scheme will be passed by the Derby Council and every other council in the realm. Indeed, the certainty of a pension following faithful service lies at the very foundation of efficient and careful asylum management. There are in this asylum 430 patients. Last year the recoveries reached 39 per cent. of the admissions, and the deaths were 10·3 per cent. on the average number resident. The weekly maintenance rate was 10s., but the Unions were charged 9s. The committee have "every reason for confidence in Dr. Lindsay," and the commissioners report that they "cannot do otherwise than remark favourably upon the state of the wards; they are, particularly on the female side, bright, clean, and comfortable." The Commissioners, however, quite properly complain that the profits on the out-county patients have been diverted from improvements and additions. We notice with surprise that there is no hospital for the treatment of infectious diseases; no mess-rooms for the attendants; that the dining-hall is overcrowded, and the single rooms deficient in number.

### NORWICH CITY ASYLUM.

While many of our county asylums err on the side of largeness, most of the borough asylums suffer from just the opposite condition. Here we have only 245 patients in residence. The year's admissions reached 59, and the deaths were 25. These give respectively per centages of 39·65 and 8·09, both calculated in the usual method. The weekly cost of maintenance stands at a fraction under 9s. 3½d. Considering the number of patients this is low, and it is creditable to Dr. Harris, provided it is not a result of undue economy, of which there is no evidence. In fact, we read that the leave of absence of all the nurses has been doubled, and their emoluments and comforts increased. A brass band has been organised among the male attendants. Dr. Harris trusts this will be productive of "harmony in feeling and work." We hope he does not mean to perpetrate a pun. An assistant medical officer has been appointed. The Commissioners in Lunacy tell us that the patients were well dressed, quiet, and contented, and that a good dinner was served in the hall, at which the utmost order prevailed.

